# Higher dietary fat quality is associated with lower anxiety score in women: a cross-sectional study

**DOI:** 10.1186/s12991-020-00264-9

**Published:** 2020-02-26

**Authors:** Fatemeh Fatemi, Fereydoun Siassi, Mostafa Qorbani, Gity Sotoudeh

**Affiliations:** 1 Department of Community Nutrition, School of Nutritional Sciences and Dietetics, Tehran University of Medical Sciences, Hojatdost street, Naderi street, Keshavarz Blv., Tehran, Iran; 2 Non-Communicable Diseases Research Center, Alborz University of Medical Sciences, Karaj, Iran; 3 Chronic Diseases Research Center, Endocrinology and Metabolism Population Sciences Institute, Tehran University of Medical Sciences, Tehran, Iran

**Keywords:** Anxiety disorder, Dietary fat quality, Fatty acids

## Abstract

**Background:**

The relationship between anxiety and dietary fat quality (DFQ) has not been well studied. The aim of this study was to investigate the relationship between anxiety disorder and fatty acids’ intake in women.

**Methods:**

This cross-sectional study included 300 women aged 18–49 attending healthcare centers. Dietary exposure was measured by a 168-item semi-quantitative food frequency questionnaire (FFQ). To determine the status of anxiety, the Depression, Anxiety, and Stress Scale (DASS) questionnaire was used. Based on the total score of anxiety, the participants were divided into two groups of without anxiety (< 8) and with anxiety (≥ 8). The relationship between fatty acids intake and odd ratio (OR) for anxiety was analyzed by simple logistic regression.

**Results:**

About 37.7% of individuals reported anxiety. After adjustment for covariates, an increase in the OR for anxiety was observed across the quintuples of saturated fatty acids (SFAs) (OR 3.17; 95% CI 1.43–7.00; *p*-trend = 0.005). In addition, higher intakes of monounsaturated fatty acids (MUFAs) (OR 0.15; 95% CI 0.05–0.44; *p*-trend = 0.001), oleic acid (OR 0.25; 95% CI 0.09–0.67; *p*-trend = 0.002), alpha-linolenic acid (ALA) (OR 0.07; 95% CI 0.02–0.23; *p*-trend < 0.001), and *n*-3:*n*-6 poly unsaturated fatty acids (PUFAs) (OR 0.56; 95% CI 0.24–1.03; *p*-trend = 0.02) were found to be related with lower OR of anxiety.

**Conclusion:**

Intake of SFAs was positively related to anxiety disorder, whereas MUFAs, oleic acid, ALA, and *n*-3: *n*-6 PUFAs intake were inversely related to anxiety score. For investigating the association of fat intake and anxiety disorder, DFQ may be a useful measure.

## Introduction

According to the World Health Organization’s (WHO) report, anxiety is one of the most common psychiatric disorders [1]. The global prevalence of anxiety has been reported 11.4% [2]. This disorder is more common in women than men [3]. The prevalence of anxiety in individuals with more than 14 years of age in Iran has been reported 15.2% (18.3% in women and 10.8% in men) [4]. Anxiety disorders are significantly related to poor social relationships [5] and physical conditions such as heart disease [6], gastrointestinal disease [7], migraine headache [8], thyroid disease [9], depression, and stress [10, 11].

Lipids play an important role in the function of neurons in the brain [12]. Neuronal cell membranes have high content of polyunsaturated fatty acids (PUFAs), especially docosahexaenoic acid (DHA), which are important for development, maintenance, and function of the nervous system. These fatty acids are involved in membrane fluidity, which is important for synaptic transmission and function of membrane proteins and neurotransmitters such as serotonin and dopamine [13–15]. Lack of *n*-3 PUFAs in the brain may lead to depression- and anxiety- associated behaviors [12]. A meta-analysis found that, compared with the control group, subjects with social anxiety disorders had lower blood levels of *n*-3 PUFAs, eicosapentaenoic acid (EPA) and DHA, and/or higher levels of *n*-6 PUFA, arachidonic acid (ARA) [16]. Lower *n*-3:*n*-6 PUFAs ratio and higher saturated fatty acids (SFAs):monounsaturated fatty acids (MUFAs) ratio may have adverse effects on the membrane fluidity [17, 18]. In mice, a reduction of MUFAs in the hippocampus resulted in alterations in cell membrane fluidity and loss of memory, learning disability, and Alzheimer's disease that causes behavioral disturbances [19, 20]. SFAs negatively affects the brain functions and increases the risk of neurological diseases [21].

Few studies have investigated the association between dietary fat quality (DFQ) and anxiety disorder. Some studies have focused on the consumption of *n*-3 PUFAs such as docosapentaenoic acid (DPA), EPA, DHA, alpha-linolenic acid (ALA), and their association with anxiety disorder. The results showed an inverse association between intake of these fatty acids and anxiety in adults [22–25]. In addition, higher consumption of fish, which is an important source of *n*-3 PUFAs, was associated with lower psychological disorders such as anxiety [24]. Due to the limitations of available cross-sectional studies, including the inherent weaknesses of design, age, and sex-specific differences and location differences, disparate results were reported. For instance, a prospective study on university graduates showed that dietary intake of *n*-3 PUFAs was not associated with anxiety disorder [26]. Another cross-sectional study in women reported no relationship between the intake of *n*-6 PUFAs and anxiety disorder [24]. Moreover, a cross-sectional study on subjects with current pure anxiety disorder did not find any association between blood *n*-6 PUFAs levels and anxiety disorders [27]. Pure anxiety is generalized anxiety disorder without depression and stress [28]. The results of experimental studies on the effects of *n*-3 PUFAs on anxiety are not also consistent. The results change depending on the administered doses and the type of fatty acids utilized in the trial. For instance, in an experimental study, consumption of 3 g/day of EPA and DHA in patients with current obsessive–compulsive disorder reduced anxiety disorder [29]. Another experimental study reported that consumption of at least 2000 mg/day *n*-3 PUFAs in adults reduced anxiety disorder [30]. However, a meta-analysis did not show beneficial effects of omega-3 supplementation on the prevention of anxiety symptoms [31].

Dietary intake may differ between countries that makes region and population-specific research of fatty acids’ intake and anxiety disorder important. The variability in fat and fatty acids’ intakes in various countries has been reported [32]. For instance, mean intake of total fat ranges from 11.1 to 46.2% E worldwide [33]. These percentages for SFAs and PUFAs are 2.9–20.9%E, and 2.8–11.3%E, respectively [33]. Even great differences in regional fat intakes have been reported in Africa, America, Asia, and Europe [32]. In Iran, consumption of total fat is approximately 22%E, with about 11.3%E coming from SFAs [34]. The proportions of MUFAs (6.8%E) and PUFAs (2.1%E) are low. Since consumption of fish is very low, vegetable oils represent the major source of *n*-3 PUFAs intake. Furthermore, due to high consumption of hydrogenated cooking fats, the intake of transfatty acids is much higher compared with many Western countries [35].

To the best of our knowledge, the relationship between anxiety disorder and some fatty acids’ intake such as MUFAs has not been investigated in human so far. As it was mentioned before, anxiety is more common in women than men. Since women are the major group of people attending healthcare centers and the sample size was not large, we restricted the study to women. Therefore, we aimed to evaluate the association between anxiety score and the DFQ in women.

## Materials and methods

### Participants

This cross-sectional study was conducted on 326 Iranian women attending healthcare centers of Amol city, from December 2017 to June 2018. Using random sampling method, 5 centers from 17 centers were selected. The frequency of women aged 18–49 years in each center was obtained. Simple sampling method was employed to select the study participants from each health center, using the proportion-to-size approach. The inclusion criteria were having 18 to 49 years of age and body mass index (BMI) 18.5–34.9 kg/m^2^. The exclusion criteria were menopause and pregnancy or lactation. Participants with diagnosed diabetes, heart disease, cancer, liver and kidney disease, hypertension, thyroid disorders, epilepsy, multiple sclerosis, depression, anxiety or stress, or drug use were excluded from the study. In addition, subjects who experienced stressful events in the last 6 months, such as divorce, love failure, loss of family, or close friends, adhering to certain diets in the past year, and smoking were not included in the study. The aim of the study was explained to the women, and written informed consent was obtained from all participants. Data were collected from each individual by face-to-face interview. General data including age, marriage status, parity, education level, job, family size, and dietary supplement intake were obtained using interview. The study was ethically approved by Ethics Committee of Tehran University of Medical Sciences.

### Anthropometric assessment

Weight was measured with the participant wearing light clothes to the nearest 0.1 kg. Height was measured in standing position, shoulders, and barefoot touching the wall to the nearest 0.5 cm [36]. Waist circumference (WC) was measured to the nearest 0.5 cm at the midpoint between the lowest rib and the top of the iliac crest in standing position [37]. Body mass index (BMI) was calculated by dividing weight in kilograms by height in squared meters (kg/m^2^).

### Physical activity assessment

Physical activity (PA) was measured using the short form of International Physical Activity Questionnaire (IPAQ) [38]. The reliability and validity of this questionnaire was assessed across 12 countries. The Spearman’s ρ for the reliability of IPAQ questionnaires has been reported around 0.8. The criterion validity had a median ρ of about 0.30, which was similar to other validation studies [39]. Participants reported times spent on strenuous, moderate, and mild physical activity over the past 7 days, and then, the values were multiplied by their metabolic equivalent (MET) quantities and the obtained numbers were summed together to calculate MET/mint/week value.

### Anxiety score assessment

To determine the status of depression, anxiety and stress, the DASS [21 items] questionnaire was used. DASS questionnaire was provided by Lovibond in 1995 [40]. The questionnaire of DASS was validated by Jafari et al. [41] in 783 Iranian medical students in 2017. The Cronbach's alpha for depression, anxiety, and stress scales have been reported 0.86, 0.76, and 0.79, respectively. In the short form of the DASS questionnaire, for each subscale of depression, anxiety, and stress, seven questions have been presented. Participants were asked to respond to each question based on to what extent that item applied to them during the last week (from 0 to 3: not at all, to some degree, to a considerable degree, and very much, respectively). At the end, the scores on the DASS-21 were multiplied by two to calculate the final score. Based on the total score of anxiety, the subjects were divided into five groups of without anxiety (0–7), mild (8–9), moderate (10–14), severe (15–19), and very severe (> 20) anxiety [40]. However, due to the limited number of cases in some groups, similar to another study [42], we simply divided participants into two groups of without anxiety (< 8) and with anxiety (≥ 8).

### Dietary intake assessment

Dietary exposure was measured by a validated 168-item semi-quantitative food frequency questionnaire (FFQ**).** This questionnaire was validated in Iran. The mean energy-adjusted reliability coefficient for nutrient intake was reported 0.6 for women. The ranges of questionnaire validity coefficients were 0.21–0.56 for protein, 0.37–0.61 for potassium, 0.38–0.50 for beta carotene, 0.31–0.95 for cholesterol, 0.21–0.55 for retinol, and 0.28–0.38 for alpha tocopherol) [43]**.**

Participants were asked to report their frequency of consumption of a given serving of each food item during the previous year, on a daily (e.g., bread), weekly (e.g., rice, meat), or monthly (e.g., fish) basis. The reported frequency for each food was converted to daily intake and was analyzed for energy and nutrient intake using the US Department of Agriculture’s (USDA) food composition tables [44]. All food intakes were analyzed for energy intake, macronutrients, and micronutrients using Nutritionist 4 software modified for Iranian foods [45]. Participants with energy intake out of predefined limits (500 kcal/day or 3500 kcal/day) were excluded (n = 26) [46]. We used healthy eating index (HEI-2015) for examining the adherence to the healthy eating guidelines [47]. In this guideline, food is divided into 13 groups: whole fruit, whole fruits without juices, whole vegetables, beans and green vegetables, whole grains, dairy, whole protein foods, seafood dishes and vegetable proteins, fatty acids, refined grains, sodium, added sugars, and SFA. Each component is based on 1000 cal. Each component was scored on a scale of 0–5 or 0–10. We excluded fatty acids and SFA when calculating the HEI score.

### Statistical analysis

Data analysis was done by Statistical Package for Social Sciences (SPSS) version 22 (SPSS Inc, Chicago). The Kolmogorov–Smirnov test was used to examine the normal distribution of variables. To compare the means of normally distributed variables between participants without anxiety and with anxiety, independent samples t test was used. Chi-square and ANOVA tests were used to determine the relationship between anxiety and qualitative and quantitative variables, respectively. All fatty acids’ intake was adjusted for energy intake using the residual method [48]. The relationship between fatty acids’ intake and odd ratio (OR) of anxiety was analyzed by simple logistic regression. In addition to the unadjusted analysis (model 1), we used multivariable models to assess the relationship between fatty acids’ intake and anxiety (model 2). In model 2, we adjusted for age, parity, job, physical activity [22], and total energy intake. These covariates have been associated with anxiety in the present and previous studies [22, 24]. *P-*values less than 0.05 were considered significant.

## Results

**Fig. 1:**
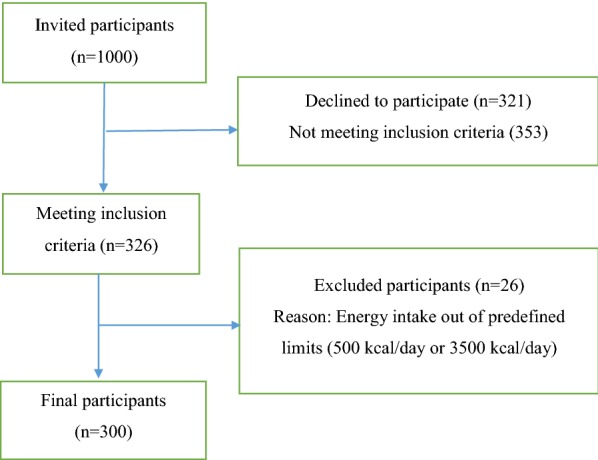
Flowchart of participants

Figure 1 shows the participant flow diagram throughout the study. A total of 1000 individuals were invited for participation in the study. Of these, 321 women declined to participate and 353 subjects did not meet eligibility criteria. About 26 individuals were excluded with energy intake out of predefined limits (500 kcal/day or 3500 kcal/day in women). Finally, 300 participants remained in the study.

The prevalence of mild, moderate, severe, and very severe anxiety was 6%, 19.7%, 7%, and 5%, respectively. About 62.3% of participants were also without anxiety. Characteristics of participants with and without anxiety are presented in Table 1. Compared with women without anxiety (< 8), participants with anxiety (≥ 8) were more employed (*p*-value = 0.005), which had lower parity (*p*-value = 0.04), higher intake of SFAs (*p*-value ≤ 0.001), lower intake of MUFAs (*p*-value = 0.01), Oleic acid (*p*-value = 0.001), and ALA (*p*-value ≤ 0.001). Other variables were not significantly different between the two groups.

**Table 1:** Characteristics of participants across anxiety status

Variable	With anxiety (score ≥ 8) *N* = 113	Without anxiety (score < 8) *N* = 187	*P*-value^a^
Age (year)^b^	30.63 ± 9.47 (18–49)	31.71 ± 8.56 (28–49)	0.3
Educational status^c^			0.9
Less than diploma	20 (17.7)	34 (18.2)	
Diploma and higher	93 (82.3)	153 (81.8)	
Marital status^c^			0.058
Single	42 (37.2)	50 (26.7)	
Married	71 (62.8)	137 (73.3)	
Job^c^			0.005
House wife	59 (52.2)	128 (68.4)	
Employed	54 (47.8)	59 (31.6)	
Dietary supplement intake^c^			0.6
Yes	54 (47.8)	84 (44.9)	
No	59 (52.2)	103 (55.1)	
Total PA^c^ (MET/min/week)			0.06
Low	63 (55.8)	116 (62)	
Moderate	23 (20.4)	46 (24.6)	
High	27 (23.9)	25 (13.4)	
Family size^b^	3.69 ± 0.93	3.58 ± 0.95	0.3
Parity^b^	0.98 ± 1.14	1.21 ± 1.21	0.04
Total energy intake^b^ (kcal/day)	2509 ± 589	2481 ± 518	0.6
HEI^b^	46.92 ± 5.95	46.93 ± 7.16	0.9
Anthropometric measures			
Height^b^	161.38 ± 5.65	161.10 ± 11.96	0.8
Body weight (kg)	66.38 ± 10.88	68.84 ± 10.45	0.053
WC (cm)	84.09 ± 11.54	85.63 ± 10.68	0.2
BMI (km/m^2^)	26.43 ± 6.92	26.59 ± 3.82	0.7
Energy-adjusted dietary intake/d^d^			
Total fat (g)	84.69 ± 17.36	82.77 ± 13.24	0.2
SFAs (g)	27.34 ± 6.34	24.69 ± 4.48	< 0.001
MUFAs (g)	25.19 ± 4.40	26.45 ± 4.48	0.01
Oleic acid (g)	22.34 ± 4.13	24.00 ± 4.32	0.001
PUFAs (g)	15.26 ± 3.74	16.12 ± 4.39	0.08
Dietary cholesterol (mg)	274.8 ± 71.8	265.2 ± 66.7	0.2
*n*-3 PUFAs (g)	0.90 ± 0.35	0.89 ± 0.32	0.8
ALA (g)	1.04 ± 0.35	1.39 ± 0.51	< 0.001
DHA (g)	0.09 ± 0.10	0.10 ± 0.11	0.3
EPA (g)	0.02 ± 0.03	0.03 ± 0.03	0.3
*n*-6 PUFAs (g)	8.82 ± 4.01	8.46 ± 3.82	0.4
*n*-3:* n*-6 PUFAs	0.10 ± 0.02	0.11 ± 0.03	0.3

Physical activity: low (less than 600 MET/min/week), moderate (600–3000 MET/min/week), and high (more than 3000 MET/min/week)

*HEI* Healthy eating index, *WC* Waist circumference, *BMI* body mass index, *PA* physical activity, *MET* metabolic equivalent, *SFAs* saturated fatty acids, *MUFAs* monounsaturated fatty acids, *PUFAs* polyunsaturated fatty acids, *ALA* alpha-linolenic acid, *DHA* docosahexaenoic acid, *EPA* eicosapentaenoic acids

^a^Chi-square test

^b^Mean and standard deviation for quantitative variables

^c^Number (%) for qualitative variables

^d^Independent *t* test

Table 2 shows the result of logistic regression analysis for odds of anxiety across the quintuples of energy-adjusted dietary fatty acids’ intake. An increase in the OR for anxiety was observed across the quintuples of SFAs (OR 3.25; 95% CI 1.51–7.02; *p*-trend = 0.001). In addition, participants included in the highest quintuple of SFAs presented higher OR for anxiety (OR 3.25; 95% CI 1.51–7.02; *p* = 0.003) as compared with participants in the first quintuple. Moreover, significant inverse associations were found between anxiety and intake of MUFAs (OR 0.37; 95% CI 0.17–0.80; *p*-trend = 0.02), oleic acid (OR 0.28; 95% CI 0.13–0.60; *p*-trend = 0.001), ALA (OR 0.08; 95% CI 0.03–0.21; *p*-trend ≤ 0.001), and *n*-3:* n*-6 PUFAs ratio (OR 0.6; 95% CI 0.31–1.37; *p*-trend = 0.02). In addition, those participants who were included in the third quintuple of PUFAs (OR 0.41; 95% CI 0.19–0.89; *p* = 0.02) and *n*-3 PUFAs (OR 0.41; 95% CI 0.18–0.92; *p* = 0.03) had lower OR as compared with participants in the first quintuple. We found no significant association between anxiety and intake of total fat (OR 1.46; 95% CI 0.67–3.15; *p*-trend = 0.1), dietary cholesterol (OR 2.04; 95% CI 0.96–4.32; *p*-trend = 0.1), DHA (OR 0.47; 95% CI 0.22–1.02; *p*-trend  = 0.2), EPA (OR 0.52; 95% CI 0.24–1.11; *p*-trend  = 0.2), and *n*-6 PUFAs (OR 1.15; 95% CI 0.54–2.45; *p*-trend  = 0.4) (Table 2, Model 1). After adjustment for age, parity, job, physical activity, BMI, and total energy intake, the positive association between anxiety and SFAs remained significant (OR 3.17; 95% CI 1.43–7.00; *p*-trend  = 0.005, Model 3). In addition, the inverse associations between anxiety and intake of MUFAs (OR 0.15; 95% CI 0.05–0.44; *p*-trend  = 0.001, Model 4), oleic acid (OR 0.25; 95% CI 0.09–0.67; *p*-trend  = 0.002, Model 4), ALA (OR 0.07; 95% CI 0.02–0.23; *p*-trend  < 0.001, Model 7), and *n*-3:* n*-6 PUFAs ratio (OR 0.56; 95% CI 0.24–1.03; *p*-trend  = 0.02, Model 5) remained significant. Furthermore, those participants who were included in the third quintuple of *n*-3 PUFAs presented lower OR for anxiety (OR 0.37; 95% CI 0.15–0.94; p = 0.03) as compared with participants in the first quintuple. After adjustment for covariates, we found no association between anxiety and intake of total fat (OR 1.20; 95% CI 0.56–2.66; *p*-trend  = 0.4; Model 2), PUFAs (OR 1.49; 95% CI 0.53–4.14; *p*-trend  = 0.1; Model 5), dietary cholesterol (OR 0.98; 95% CI 0.39–2.50; *p*-trend  = 0.4; Model 6), DHA (OR 0.59; 95% CI 0.25–1.40; *p*-trend  = 0.6; Model 7), EPA (OR 0.62; 95% CI 0.26–1.48; *p*-trend  = 0.4; Model 7), and *n*-6 PUFAs (OR 1.24; 95% CI 0.38–4.08; *p*-trend  = 0.3; Model 8) (Table 2).

**Table 2:** Odds ratio (OR) and 95% confidence intervals (CIs) for anxiety across the quintuples (Q) of energy-adjusted dietary fatty acid intake

	Q1 (*n* = 60)	Q2 (*n* = 60)	Q3 (*n* = 60)	Q4 (*n* = 60)	Q5 (*n* = 60)	*p*-trend
Total fat (non-case/case)	(43/17)	(39/21)	(35/25)	(32/28)	(38/22)	
Model 1	1.00	1.36 (0.62–2.94)	1.80 (0.84–3.86)	2.21 (1.03–4.71)	1.46 (0.67–3.15)	0.1
*P*-value		0.4	0.1	0.1	0.4	
Model 2	1.00	1.23 (0.55–2.73)	1.77 (0.79–3.96)	1.81 (0.82–3.98)	1.20 (0.56–2.66)	0.4
*P*-value		0.6	0.1	0.1	0.6	
SFAs (non-case/case)	(44/16)	(43/17)	(35/25)	(37/23)	(28/32)	
Model 1	1.00	1.08 (0.48–2.42)	1.96 (0.91–4.23)	1.66 (0.76–3.60)	3.25 (1.51–7.02)	0.001
*P*-value		0.8	40.08	0.1	0.003	
Model 3: model 2 + MUFAs, PUFAs, Trans	1.00	1.36 (0.59–3.12)	2.12 (0.96–4.69)	1.66 (0.75–3.70)	3.17 (1.43–7.00)	0.005
*P*-value		0.4	0.06	0.2	0.004	
MUFAs (non-case/case)	(29/31)	(38/22)	(39/21)	(39/22)	(42/17)	
Model 1	1.00	0.54 (0.26–1.12)	0.50 (0.24–1.04)	0.52 (0.25–1.09)	0.37 (0.17–0.80)	0.02
*P*-value		0.6	0.6	0.02	0.2	
Model 4: model 2 + SFAs, PUFAs, Trans	1.00	0.41 (0.19–0.90)	0.36 (0.16–0.82)	0.34 (0.15–0.77)	0.15 (0.05–0.44)	0.001
*P*-value		0.02	0.01	0.01	0.001	
Oleic acid (non-case/case)	(25/35)	(38/22)	(40/20)	(41/19)	(43/17)	
Model 1	1.00	0.42 (0.20–0.88)	0.34 (0.16–0.73)	0.33 (0.15–0.69)	0.28 (0.13–0.60)	0.001
*P*-value		0.02	0.005	0.004	0.001	
Model 4: model 2 + SFAs, PUFAs, Trans	1.00	0.43 (0.20–0.92)	0.33 (0.15–0.72)	0.28 (0.12–0.64)	0.25 (0.09–0.67)	0.002
*P*-value		0.03	0.006	0.002	0.006	
PUFAs (non-case/case)	(32/28)	(39/21)	(44/16)	(30/30)	(42/18)	
Model 1	1.00	0.61 (0.29–1.28)	0.41 (0.19–0.89)	1.14 (0.55–2.34)	0.49 (0.23–1.03)	0.3
*P*-value		0.1	0.02	0.7	0.06	
Model 5: model 2 + SFAs, MUFAs, Trans	1.00	0.96 (0.41–2.23)	1.71 (0.72–4.05)	2.35 (0.99–5.58)	1.49 (0.53–4.14)	0.1
*P*-value		0.9	0.2	0.052	0.4	
Dietary cholesterol (non-case/case)	(42/18)	(34/26)	(41/19)	(38/22)	(32/28)	
Model 1	1.00	1.78 (0.84–3.78)	1.08 (0.49–2.34)	1.35 (0.63–2.89)	2.04 (0.96–4.32)	0.1
*P*-value		0.1	0.8	0.4	0.06	
Model 6: model 2 + SFAs, PUFAs, MUFAs, Trans	1.00	1.91 (0.83–4.41)	0.92 (0.38–2.22)	0.93 (0.38–2.20)	0.98 (0.39–2.50)	0.4
*P*-value		0.2	0.5	0.6	0.7	
*n*-3 PUFAs (non-case/case)	(36/24)	(35/25)	(47/13)	(32/28)	(37/23)	
Model 1	1.00	1.07 (0.51–2.21)	0.41 (0.18–0.92)	1.31 (0.63–2.70)	0.93 (0.44–1.94)	0.9
*P*-value		0.8	0.03	0.4	0.8	
Model 7: model 2 + SFAs, MUFAs, Trans,* n*-6	1.00	1.05 (0.46–2.40)	0.37 (0.15–0.94)	0.92 (0.38–2.26)	0.66 (0.21–2.04)	0.4
*P*-value		0.8	0.03	0.8	0.4	
ALA (non-case/case)	(24/36)	(35/25)	(28/32)	(46/14)	(54/6)	
Model 1	1.00	0.44 (0.21–0.92)	0.73 (0.35–1.51)	0.18 (0.08–0.40)	0.08 (0.03–0.21)	< 0.001
*P*-value		0.02	0.3	< 0.001	< 0.001	
Model 7: model 2 + SFAs, MUFAs, Trans,* n*-6	1.00	0.34 (0.15–0.79)	0.59 (0.25–1.39)	0.14 (0.05–0.37)	0.07 (0.02–0.23)	< 0.001
*P*-value		0.6	0.6	0.1	< 0.001	
DHA (non-case/case)	(34/26)	(38/22)	(39/21)	(32/28)	(44/16)	
Model 1	1.00	0.75 (0.36–1.57)	0.70 (0.33–1.47)	1.14 (0.55–2.35)	0.47 (0.22–1.02)	0.2
*P*-value		0.4	0.3	0.7	0.05	
Model 7: model 2 + SFAs, MUFAs, Trans,* n*-6	1.00	0.77 (0.34–1.73)	0.94 (0.41–2.15)	1.29 (0.57–2.90)	0.59 (0.25–1.40)	0.6
P-value		0.5	0.8	0.5	0.2	
EPA (non-case/case)	(33/27)	(39/21)	(37/23)	(36/24)	(42/18)	
Model 1	1.00	0.65 (0.31–1.37)	0.76 (0.36–1.57)	0.81 (0.39–1.68)	0.52 (0.24–1.11)	0.2
*P*-value		0.2	0.4	0.5	0.09	
Model 7: model 2 + SFAs, MUFAs, Trans, *n*-6	1.00	0.63 (0.27–1.44)	0.93 (0.41–2.12)	0.78 (0.35–1.75)	0.62 (0.26–1.48)	0.4
*P*-value		0.2	0.8	0.5	0.2	
*n*-6 PUFAs (non-case/case)	(40/20)	(40/20)	(35/25)	(34/26)	(38/22)	
Model 1	1.00	1.00 (0.46–2.13)	1.42 (0.68–3.00)	1.52 (0.72–3.20)	1.15 (0.54–2.45)	0.4
*P*-value		1.0	0.3	0.2	0.7	
Model 8: model 2 + SFAs, MUFAs, Trans, *n*-3	1.00	0.89 (0.37–2.10)	1.56 (0.65–3.71)	1.61 (0.65–4.00)	1.24 (0.38–4.08)	0.3
*P*-value		0.7	0.3	0.2	0.7	
*n*-3:* n*-6 (non-case/case)	(34/26)	(33/27)	(33/27)	(47/13)	(40/20)	
Model 1	1.00	1.07 (0.5–2.2)	1.07 (0.5–2.2)	0.3 (0.16–0.80)	0.6 (0.31–1.37)	0.02
*P*-value		0.85	0.85	0.01	0.26	
Model 5: model 2 + SFAs, MUFAs, Trans	1.00	0.97 (0.42–2.23)	0.94 (0.41–2.17)	0.36 (0.15–0.88)	0.56 (0.24–1.03)	0.02
*P*-value		0.9	0.9	0.02	0.1	

Model 1: Crude

Model 2 Adjusted for age, physical activity, total energy intake, job, body mass index, and parity

*SFAs* saturated fatty acids, *MUFAs* monounsaturated fatty acids, *PUFAs* polyunsaturated fatty acids, *ALA* alpha-linolenic acid, *DHA* docosahexaenoic acid, *EPA* eicosapentaenoic acids

## Discussion

In the present study, we investigated the relationship between anxiety disorder and DFQ in women. The result of this study suggested that higher intake of SFAs was associated with increased anxiety score. In addition, intake of MUFAs, oleic acid, ALA, and *n*-3:* n*-6 PUFAs ratio was associated with lower odds of anxiety. Our study showed no significant association between intake of DHA, EPA, *n*-6 PUFAs, cholesterol, and total fat with anxiety disorder.

Our study showed that higher SFAs intake was associated with higher anxiety score. Similarly, a randomized crossover-design study in university students showed that SFAs intake is positively related to anxiety score [49]. In our study, a higher intake of MUFAs and oleic acid was related to lower anxiety score. To the best of our knowledge, no study has reported the association of MUFAs with anxiety disorder. In addition, we showed an inverse association between *n*-3:*n*-6 PUFAs ratio and anxiety score. Similar to our study results, a prospective cohort study showed a positive significant association between lower ratio of *n*-3: *n*-6 PUFAs and anxiety disorder [50]. Although no association was found between DHA, EPA, and *n*-6 PUFAs with anxiety score in the present study, the data from some cross-sectional studies have shown an inverse relationship between intake of DHA and EPA with anxiety in adults [22–24]. In addition, some experimental studies have shown that intake of EPA and DHA reduces anxiety disorder [29, 51]. However, a prospective study did not find any relationship between intake of *n*-3 PUFAs and anxiety disorder [26]. In addition, a cross-sectional study showed that dietary intake of *n*-6 PUFAs is not related with anxiety [24]. Contrary to our findings, an experimental study on adults showed beneficial effect of low-fat diet in comparison with a high-fat diet on psychological mood states [52]. The disparity between the findings might be explained by the differences in study design, sample size, characteristics of study participants, and geographic differences. Some biological mechanisms explain the effect of fatty acids in psychiatric disorders; these include regulation of corticotropin-releasing factor, the hypothalamic-pituitary-adrenocortical axis, increased serotonergic neurotransmission, alterations in dopaminergic function, and improved cerebral blood flow [53–55]. In addition, fatty acids have an effect on receptor function, signal transmission, and neurotransmitter reuptake [26]. A few studies have examined the relationship between fatty acids’ intake and anxiety, especially in a general population. The current study was adjusted for several important confounders that are known to affect anxiety disorder and DFQ. In addition, women in our study were not informed of their anxiety status. When individuals are aware of their anxiety disorder, they might change their food intake or dietary intake report. However, the study has some limitations. First, FFQ has a potential recall bias. Respondents are requested to report their intake retrospectively, which may affect their reporting. Recall bias increases with recall periods longer than 7 days [56]. The FFQ used in the present study was excessively long and required recall of up to 1 year, which may affect the report of dietary intake in the participants. In addition, it is difficult for respondents to answer the questions regarding usual frequency of intake and usual portion size, and are thus prone to measurement error [56]. Second, the study design was cross-sectional, which cannot interpret a cause-and-effect relationship. Third, it is possible that experiencing anxiety might have resulted in lifestyle modification including lower dietary intake or altered dietary choices, which could result in potential bias. Fourth, regardless of adjusting for many confounding variables in the analysis, residual confounding together with unmeasured confounders may have affected the results. Fifth, as the study population was women who attended the healthcare centers, we cannot generalize our results to all women in this age group. In addition, the findings might not be generalized to men and populations with ethnic and cultural differences.

## Conclusion

Based on the results of the present study, an inverse association was found between intakes of MUFAs, oleic acid, ALA, and *n*-3:*n*-6 PUFAs ratio with odds of anxiety. In addition, higher intake of SFAs was associated with higher anxiety score. The study analysis showed that overall DFQ may be important in anxiety disorder. These results may help to develop nutritional interventions and dietary guidelines to prevent or reduce anxiety in women. Nevertheless, experimental studies are required to evaluate the effect of DFQ on anxiety disorder.

## Data Availability

The data used to support the findings of this study are available from the corresponding author.
